# Unique characteristics of autoantibodies targeting MET in patients with breast and lung cancer

**DOI:** 10.1172/jci.insight.187392

**Published:** 2025-05-22

**Authors:** Michal Navon, Noam Ben-Shalom, Maya Dadiani, Michael Mor, Ron Yefet, Michal Bakalenik-Gavry, Dana Chat, Nora Balint-Lahat, Iris Barshack, Ilan Tsarfaty, Einav Nili Gal-Yam, Natalia T. Freund

**Affiliations:** 1 Department of Microbiology and Clinical Immunology, Faculty of Medical and Health Sciences, Tel Aviv University, Tel Aviv, Israel.; 2 Cancer Research Center, Sheba Medical Center, Ramat Gan, Israel.; 3 Center for Infectious Disease and Vaccine Research, La Jolla Institute for Immunology, La Jolla, California, USA.; 4 Department of Pathology, Sheba Medical Center, Ramat Gan, Israel.; 5 Department of Pathology, Faculty of Medical and Health Sciences, Tel Aviv University, Tel Aviv, Israel.; 6 Institute of Breast Oncology, Jusidman Cancer Center, Sheba Medical Center, Ramat Gan, Israel.

**Keywords:** Autoimmunity, Immunology, Adaptive immunity, Cancer

## Abstract

The presence of B cells in tumors is correlated with favorable prognosis and efficient response to immunotherapy. While tumor-reactive antibodies have been detected in several cancer types, identifying antibodies that specifically target tumor-associated antigens remains a challenge. Here, we investigated the antibodies spontaneously elicited during breast and lung cancer that bind the cancer-associated antigen MET. We screened patients with lung (*n* = 25) and breast (*n* = 75) cancer and found that 13% had antibodies binding to both the recombinant ectodomain of MET, and the ligand binding part of MET, SEMA. MET binding in the breast cancer cohort was significantly correlated with hormone receptor–positive status. We further conducted immunoglobulin sequencing of peripheral MET-enriched B cells from 6 MET-reactive patients. The MET-enriched B cell repertoire was found to be polyclonal and prone to non-IgG1 subclass. Nine monoclonal antibodies were cloned and analyzed, and these exhibited MET binding, low thermostability, and high polyreactivity. Among these, antibodies 87B156 and 69B287 effectively bound to tumor cells and inhibited MET-expressing breast cancer cell lines. Overall, our data demonstrate that some patients with breast and lung cancer develop polyreactive antibodies that cross-react with MET. These autoantibodies have a potential contribution to immune responses against tumors.

## Abstract 2

Autoantibodies against the oncoreceptor MET were detected in breast and lung cancer patients, exhibiting anti-cancer effects on tumor cells along with polyreactivity and low thermostability.

## Introduction

The tumor microenvironment (TME) is populated by infiltrating immune cells, including B cells, and their presence has been related to a positive prognosis and better responsiveness to immune checkpoint blockade (ICB) (1, 2). Within tumors, B cells can reside in tertiary lymphoid structures (TLS), which are germinal center–like immune niches where they can take up and present antigens to T cells and differentiate into antibody-secreting cells (ASCs) (3–5). In addition, studies have shown that anticancer B cells and antibodies circulate in cancer patients’ blood (6–8). Recent studies have characterized intratumoral antibodies, revealing key features including high clonality, class switching, autoreactivity, and high frequencies of somatic hypermutation (SHM) — characteristics that suggest an antigen-directed immune response (4, 9).

B cells develop in the bone marrow, where they undergo positive and negative selection. B cells expressing self-reactive B cell receptors (BCRs) are supposed to be removed during B cell development, to prevent autoimmune responses. Yet, a substantial frequency of the mature repertoire express BCRs targeting self-proteins (10). Such B cells exist by escaping either central tolerance checkpoints during development or peripheral tolerance checkpoints following affinity maturation against non-self targets (11). Antibody production is typically initiated when a B cell encounter’s its cognate antigen, followed by class switch recombination and affinity maturation (12). This process may also generate self-reactive B cells that produce autoantibodies, which in some cases can be detrimental (13, 14). However, not all autoantibodies are harmful. As such, immune suppression and dysregulation occurring during cancer can result in activation of B cells, resulting in the production of autoreactive autoantibody responses (15). Both the function of these B cells and the role of their immunoglobulins in tumor progression remain unclear.

Studies have reported autoantibodies against cancer-associated antigens in patients with various types of cancer (16, 17). In the TME of patients with ovarian cancer, ASCs produce antibodies directed against matrix metalloprotease-14 (MMP14), an enzyme that is highly expressed on the tumor cell surface (18). An appealing group of naturally elicited anti-cancer antibodies includes immunoglobulins targeting tumor-associated growth factor receptors. As such, preexisting anti-HER2 and anti-EGFR serum antibodies were found in patients, and these antibodies correlate with successful therapy outcomes and positive prognosis (19, 20). Other studies describing B cell responses in the circulation during cancer development generated unclear conclusions regarding the correlation of these antibodies with cancer progression. On one hand, persistent plasmablast responses in the circulation have been linked to anti-tumor antibody responses in patients with breast cancer, and the secreted antibodies were shown to induce anti-cancer effects and cancer regression in mice (7). On the other hand, serum antibody reactivity has also been correlated with more advanced disease in patients with melanoma (21). Overall, since defined tumor-related antigens are scarce, isolation of B cells and antibodies that target specific tumor-associated antigens and the functional characterization of these antibodies remains a major goal and a substantial challenge.

MET is a receptor tyrosine kinase activated by hepatocyte growth factor (HGF) (22). Similarly to other growth factor receptors, MET functions physiologically during embryogenesis and tissue repair (23). During cancer, aberrant MET activation promotes tumorigenesis and metastasis (24, 25). MET overexpression is observed in breast, lung, gastric, and colorectal cancers and correlates with bad prognosis (24, 26, 27). However, whether MET autoantibodies are produced in patients with cancer, and what is their function, are currently unknown. Here, we used MET as a model to study autoantibody responses in patients with cancer. We identified circulating MET-binding B cells and autoantibodies in patients with both breast and lung cancer. These autoantibodies were largely polyreactive and were not specific to MET. Nevertheless, they exhibited anti-tumor activity, indicating their potential role in the immune response against cancer.

## Results

### Antibodies binding MET are detected in a subset of patients with breast and lung cancer.

Our primary question was whether patients with cancer develop antibodies binding the oncoreceptor MET, and if so, to characterize this response at serological and B cell levels. To this end, we collected sera from 100 patients diagnosed with cancer (75 with breast cancer and 25 with lung cancer, Table 1). Most of the patients had metastatic disease (49 breast cancer and 17 lung cancer), 26 patients with breast cancer had early-stage disease and were receiving neoadjuvant therapy, and 8 patients with nonmetastatic lung cancer were undergoing chemoradiation therapy (Supplemental Table 1; supplemental material available online with this article; https://doi.org/10.1172/jci.insight.187392DS1).

**Figure 1:**
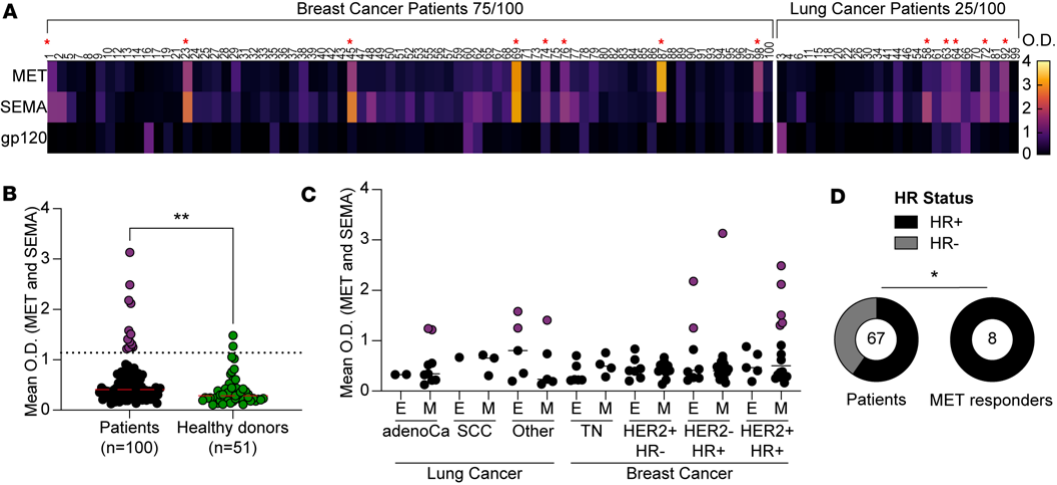
Anti-MET response in a cohort of 100 patients with cancer. (**A**) Heatmap representing cancer patient sera binding as detected by ELISA. Each column represents 1 donor (donor IDs are given on top). The left side of the heatmap presents breast cancer donors, while the right side presents lung cancer donors. The rows are based on raw OD_650_ values at a serum dilution of 1:50 for MET, SEMA, and gp120. Color code is given on the right of the figure, where light yellow indicates binding, and dark purple indicates lack of binding. The 13 identified patients who responded to MET are marked with red asterisks. (**B**) Mean OD of MET and SEMA binding of cancer patients (black/purple, *n* = 100) and non-cancer donors (green, *n* = 51). The dashed line indicates the 95th percentile of healthy donors’ sera scores (set as the threshold for defining anti-MET positive response). The 13 patients who responded to MET are colored in purple. (**C**) Mean OD of MET and SEMA binding for each patient, subdivided by cancer type, subtype (adenoCa = adenocarcinoma, SCC = squamous cell carcinoma, TN = triple negative), and stage (E = early, M = metastasis). The 13 patients who responded to MET are indicated in purple. (**D**) Pie charts showing the tumor hormonal receptor status (HR^+^ versus HR^–^) in 67 patients with breast cancer who did not respond to MET (left pie), as opposed to the 8 patients with breast cancer who responded to MET (right pie). *P < 0.05; **P < 0.01 by Kolmogorov-Smirnov test (**B**) or Fisher’s exact test (**D**).

MET is recognized as a tumor antigen in both breast and lung cancers (28, 29), and its expression correlates with poor prognosis (28, 30). We first tested whether our cohort exhibits serum antibodies that are capable of binding MET. Patient sera were screened, by ELISA, against the recombinantly expressed 908–amino acid extracellular portion of MET, and the 536–amino acid ligand-binding domain of MET, known as SEMA (Supplemental Figure 1). HIV-1 gp120 antigen, produced in the same system and carrying the same tags as MET and SEMA, was used as a control antigen (all donors were HIV-1 negative). Patient sera bound MET and SEMA, with a significant correlation between serum binding to the 2 molecules (*r* = 0.5005, *P* = 0.0344; Figure 1A and Supplemental Figure 2A), while there was no correlation between binding to MET and binding to gp120 (*r* = 0.09411, *P* = 0.7103; Supplemental Figure 2A). Compared with healthy donors (*n* = 51), patients showed significantly higher binding to MET and SEMA (*P* = 0.0094 and *P* < 0.0001, respectively), while binding to gp120 was similar (Supplemental Figure 2B). To assess overall MET response, we calculated the mean OD values for MET and SEMA binding. This analysis also showed significantly higher binding in patients compared with healthy donors (*P* = 0.0033, Figure 1B). MET response was defined as any score above the 95th percentile of the healthy donor cohort scores. According to this threshold, 13 patients with cancer exhibited serum binding to MET: 8 patients with breast cancer (10.6%) and 5 patients with lung cancer (20%) (Figure 1A, indicated by red asterisks, and Supplemental Table 1). For 4 of the 13 patients with serological MET responses (P1, P45, P76, and P72), tumor tissue sections were available, along with 3 additional sections from patients without serum MET binding. Surprisingly, in this small number of samples, we did not observe any correlation between MET expression and MET serum responses (Supplemental Figure 2C).

Our cohort included both metastatic and nonmetastatic breast and lung cancer donors, with a limited number for each subtype (Figure 1C). This heterogeneity limited our ability to identify specific patient features associated with anti-MET response. We therefore focused on patients with breast cancer (*n* = 75), where 8 patients with anti-MET response were identified. Interestingly, all 8 patients with breast cancer responding to MET were hormone receptor–positive (HR^+^), and HR^+^ status was significantly associated with anti-MET response (*P* = 0.0448, Figure 1, C and D). Moreover, when focusing on the 32 patients with metastatic breast cancer who are HR^+^, and for whom survival data were available, a trend of improved survival within the MET-binding patients was identified, although it was not statistically significant (*P* = 0.28, Supplemental Figure 2D). For patients with lung cancer, we assessed the presence of EGFR mutations and found no clear association with anti-MET responses, as only 2 patients in the cohort had EGFR mutations, one of whom exhibited a response to MET.

### Peripheral MET B cell repertoire is polyclonal with less IgG1.

**Figure 2:**
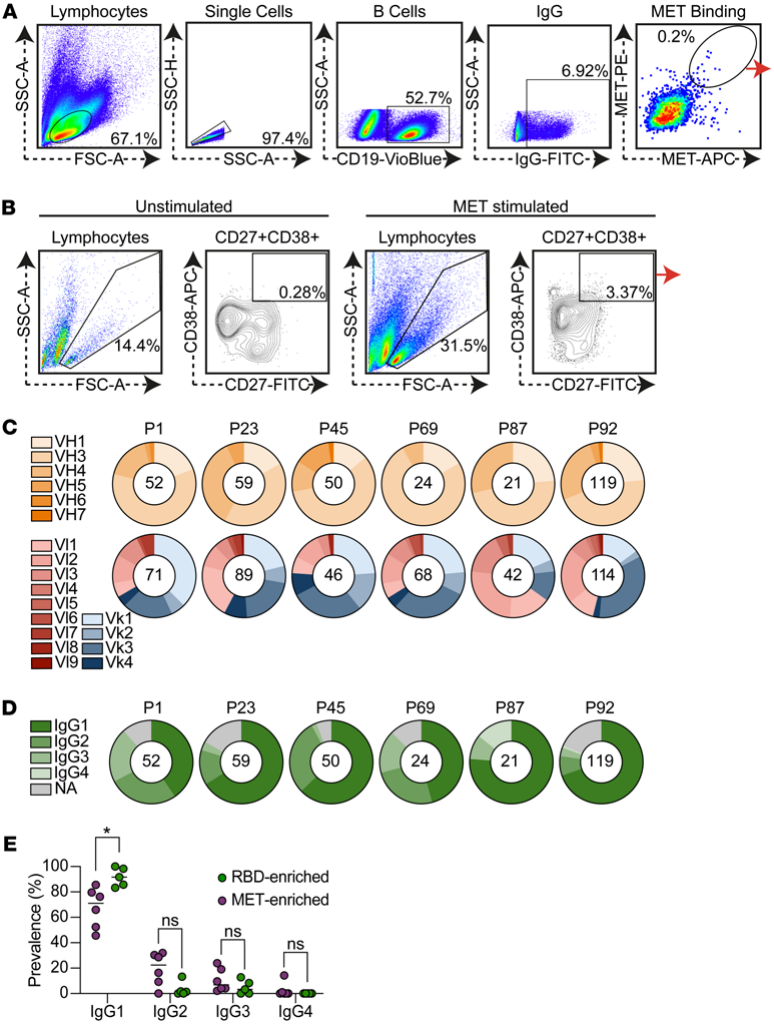
Isolation and sequencing of MET-binding B cells. (**A**) Gating strategy for isolation of MET-binding B cells directly from PBMCs: lymphocytes → singlets → B cells (CD19^+^) → IgG^+^ → MET^+^ (double fluorophore staining). The last gate was single-cell sorted. (**B**) Gating strategy for isolation of MET-binding B cells following B cell in vitro culturing with MET: lymphocytes → CD38^hi^CD27^hi^. The last gate was single-cell sorted. (**C**) Pie charts representing the V_H_ and V_L_ usage of MET-enriched B cells. Each pie chart represents 1 patient (indicated on top), the numbers in the middle of the pies represent the total sequences recovered, and slices are proportional to the representation of specific V_H_ or V_L_ gene. (**D**) Pie charts representing the IgG subclass distribution of MET-enriched B cells. Each pie chart represents 1 patient (indicated on top), the numbers in the middle of the pies represent the total sequences recovered, and slices are proportional to the representation of specific subclass. (**E**) IgG subclass prevalence of MET-binding B cells from 6 patients with cancer compared to RBD-binding B cells from 5 COVID-19–convalescent patients. *P < 0.05 by Mann-Whitney test.

To gain more insights into MET-targeting B cells, we analyzed the MET-reactive B cell repertoire by sequencing B cells enriched for MET binding. To confirm serum MET activity, IgG was purified from the serum, and the ELISA was repeated with quantified IgG. We then focused on 6 cancer donors with the highest IgG binding to MET: P1, P23, P45, P69, P87, and P92 (Supplemental Figure 2E, shaded area; P72 was omitted due to limited sample availability). For these donors, peripheral blood mononuclear cells (PBMCs) were isolated from whole blood and MET-binding B cells were sorted using fluorescently tagged MET baits (31). Approximately 0.2% of the IgG^+^CD19^+^ B cells exhibited positive binding to MET, and these were single-cell sorted (Figure 2A). In accordance with previous results (8), patients with cancer in our study exhibited low frequencies of IgG^+^ B cells, with an average of 5.56% (SD = 2.26) from total CD19^+^ cells (Supplemental Figure 3), resulting in a low number of MET-binding B cells. To increase the number of MET-binding B cells, we took a parallel approach where prior to sorting, B cells were cultured with recombinant MET, resulting in activation and expansion of MET-reactive B cells through their antibodies (identified by CD38^hi^ and CD27^hi^). This technique was previously demonstrated to activate and increase the frequency of rare, antigen-specific B cells, without inducing SHM or class switching (32, 33). These cells were then single-cell sorted (Figure 2B). Using both strategies, we collected 995 B cells, resulting in 325 heavy chain and 430 light chain sequences, as detailed in Supplemental Table 2. Analysis of the immunoglobulin sequences of MET-enriched B cells revealed a diverse repertoire of V_H_ and V_L_ genes (Figure 2C). V_H_3 was the most frequent V_H_ gene across all patients. No V_H_J_H_ combinations were shared across all patients (Supplemental Figure 4A). Kappa light chain was more frequent than lambda in 5 out of 6 studied patients (Figure 2C).

MET B cells exhibited lower levels of IgG1 subclass compared with what was reported for other antigens (34), or compared with what we previously reported for B cells from SARS-CoV-2–convalescent donors without cancer (isolated using the same protocols against the SARS-CoV-2 receptor binding domain [RBD]; refs. 32, 35) (Figure 2, D and E). MET-enriched B cells exhibited 67.5% IgG1 compared with 91.8% in RBD-enriched B cells from donors without cancer (*P* = 0.0345, Figure 2E). To rule out any bias that could have been introduced by our culturing technique, we compared the frequency of IgG1 in MET B cells recovered from both methods (direct sorting versus culturing prior to sorting) and found no differences, indicating that the dominance of non-IgG1 is not protocol related (Supplemental Figure 4B).

### Patient-derived monoclonal antibodies bind tumor cells and are polyreactive.

**Figure 3:**
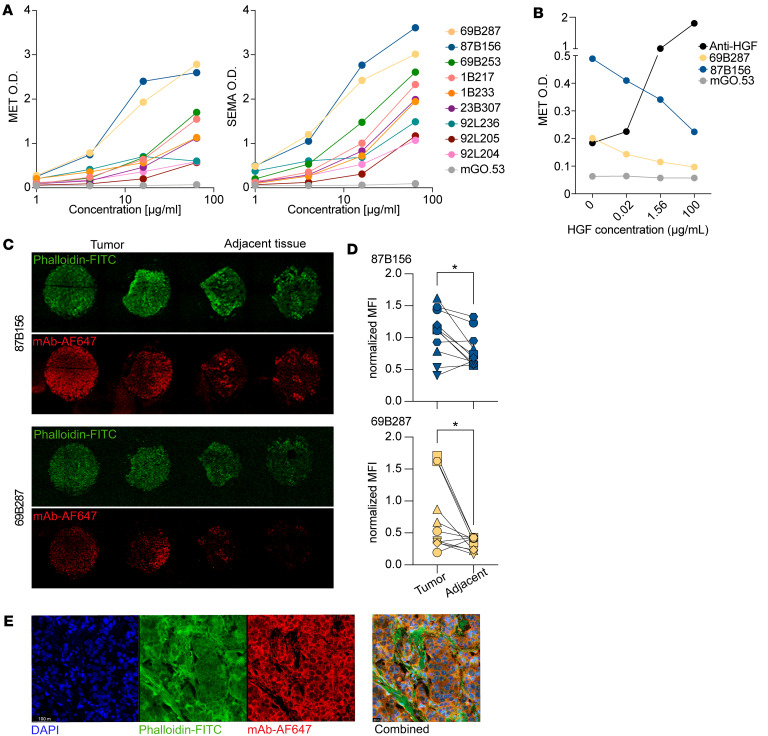
MET-binding mAbs cloned from patients with cancer. (**A**) Binding of anti-MET mAbs to MET (left panel) and to SEMA (right panel), as detected by ELISA. mGO.53 serves as an isotype control (10). The legend on the right side indicates mAb IDs. (**B**) Competition ELISA of anti-MET mAbs (69B287, 87B165, and mGO.53) with HGF. OD_650_ values represent the binding of mAbs to MET following incubation with varying HGF concentrations (100, 1.56, 0.02, and 0 μg/mL). A commercial anti-HGF mAb (5 μg/mL) was included to assess HGF binding to MET. (**C**) Representative images of tumor tissue (left) and healthy adjacent tissue (right) stained with phalloidin and 87B156 (top) or 69B287 (bottom) mAb. (**D**) Quantification and normalization of 87B156 (top) or 69B287 (bottom) mAb to phalloidin between tumor (*n* = 11) and healthy tissues (*n* = 11). See also Supplemental Figure 5. *P < 0.05 by Wilcoxon’s test. (**E**) Representative images of tumor tissue stained with DAPI, phalloidin conjugated to FITC, mAb conjugated to Alexa Fluor 647, and the combined image with all staining. Scale bars: 100 μm (left 3 images) and 50 μm (combined).

The properties of MET-binding antibodies were investigated by expressing representative monoclonal antibodies (mAbs). Nine mAbs, 69B287, 87B156, 69B253, 1B277, 1B233, 23B307, 92L236, 92L205, and 92L204, demonstrated a detectable binding to MET and SEMA by ELISA (Figure 3A). mAbs 69B287 and 87B156, which exhibited the highest binding among the 9 tested mAbs, competed with HGF for MET binding (Figure 3B). These mAbs also bound to a human breast cancer tissue array. The antibody staining colocalized to the cell membrane and showed significantly lower binding to the adjacent healthy tissue and significantly higher binding compared with the isotype control antibody, mGO.53 (10) (Figure 3, C–E, and Supplemental Figure 5).

**Figure 4:**
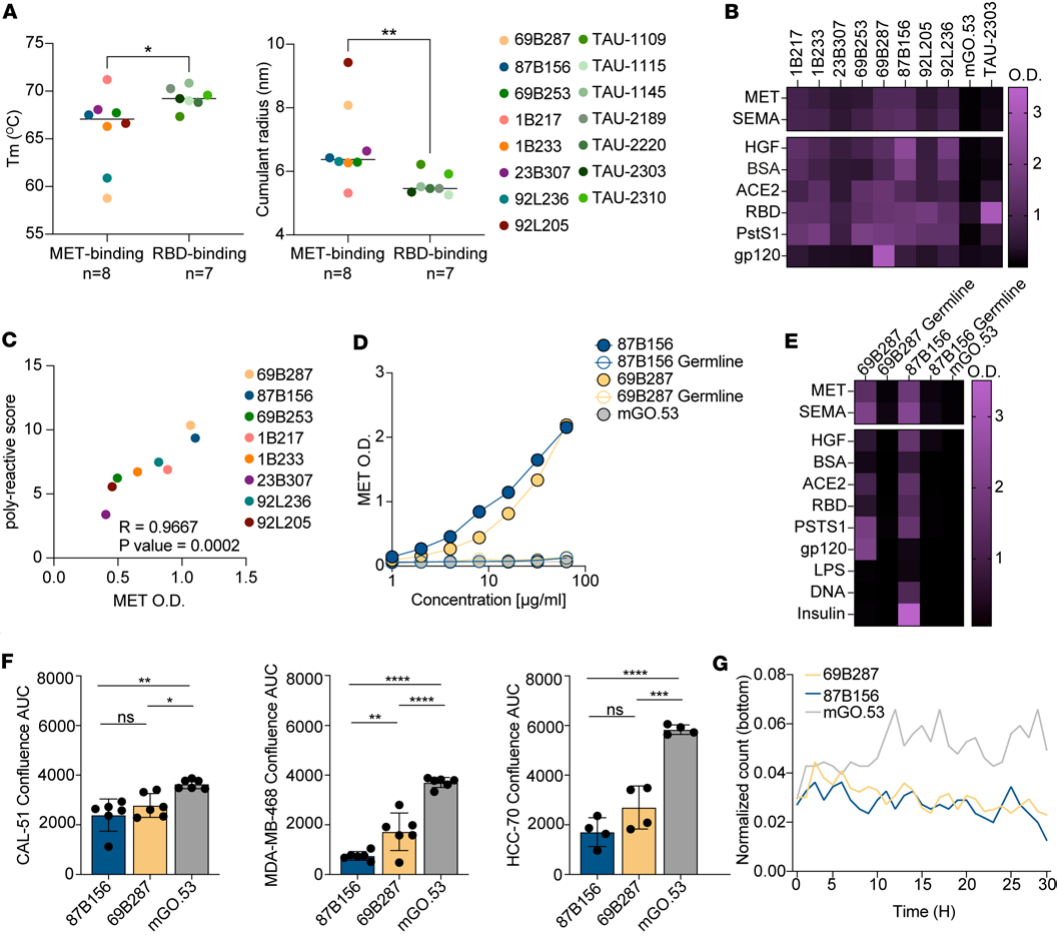
MET-binding antibodies are polyreactive. (**A**) Comparison between denaturation midpoint (left) and cumulant radius (right) of MET-binding mAbs (*n* = 8) and RBD-binding mAbs (*n* = 7). (**B**) Heatmap representing binding to non-MET proteins as detected by ELISA. Each column represents 1 mAb, and each row represents a single antigen. Color code is given on the right of the figure. mGO.53 is an isotype nonbinding control and TAU-2303 is an anti-RBD mAb that is not polyreactive (35). (**C**) Correlation between MET binding and polyreactive score, calculated by the average of all non-MET antigens at OD_650_. (**D**) Binding of 69B287, 87B156, their predicted germline versions, and germline-mutated chimeric versions to MET as detected by ELISA. (**E**) Heatmap representing binding by ELISA of 69B287, 87B156, their predicted germline versions, and germline-mutated chimeric versions to non-MET proteins. (**F**) Calculated AUC of the confluence measured by Incucyte of CAL-51, MDA-MB-468, and HCC70 breast cancer cell lines over time, incubated with each mAb (87B156, 69B287, and mGO.53), with 4–6 replicates for each condition. Data presented as mean ± SD. *P < 0.05; **P < 0.01; ***P < 0.001; ****P < 0.0001 by 1-way ANOVA with Tukey’s multiple-comparison post hoc test. (**G**) Quantification of chemotactic cell migration and invasion. A total of 5000 CAL-51 cells were seeded in the upper chamber of each well in a 96-well chemotaxis microplate and treated with 100 μM mAbs (87B156, 69B287, and mGO.53). EGF (1 μg/mL) was added to the lower chamber of all wells as a chemoattractant. Cell migration was monitored using live imaging, and the number of migrated CAL-51 cells was quantified. Migration counts were normalized to the initial cell number, with 3 replicates for each condition.

To characterize the mAbs biochemically, we used dynamic light scattering (DLS). MET-binding mAbs showed lower thermostability and higher cumulant radius compared with control mAbs from SARS-CoV-2–infected healthy donors produced on the same backbone (35) (Figure 4A; 92L204 was excluded from further analyses due to low production yields). A higher cumulant radius indicates increased molecular size and aggregate formation. In antibodies, this indicates greater exposure of hydrophobic regions and structural flexibility, which are molecular features often associated with polyreactivity (36, 37). Such polyreactivity has been previously documented for anti-cancer antibodies (21). In agreement with this, MET-binding mAbs bound to non-MET proteins, and this cross-reactivity correlated with their MET binding as measured by ELISA (Figure 4, B and C). Both polyreactivity and MET binding were a result of affinity maturation, as both germline versions of 69B287 and 87B156 did not bind MET as well as the mutated antibodies, or any of the tested non-MET proteins (Figure 4, D and E). Notably, 87B156 also recognized LPS, insulin, and DNA, whereas 69B287 and both germline antibodies did not bind these antigens (Figure 4E) (18). These results suggest that MET binding by both mAbs is largely dependent on SHM.

### MET-binding antibodies exhibit an inhibitory effect on tumor cells in culture.

We next aimed to assess the impact of our mAbs on 3 human MET-expressing breast cancer cell lines: CAL-51, MDA-MB-468, and HCC70 (38, 39) (Supplemental Figure 6A). The cells were incubated with either 69B287 or 87B156 and we monitored cell confluence using live cell imaging for 60 hours. All 3 cell lines grew more slowly in the presence of 69B287 or 87B156, an effect that was not observed when the cells were cultured with the isotype control (Figure 4F and Supplemental Figure 6B). We next assessed the impact of 69B287 and 87B156 on cell invasion using a chemotaxis assay (Supplemental Figure 6C). Notably, treatment with 69B287 or 87B156 significantly inhibited the ability of CAL-51 cells to migrate across the membrane, as opposed to isotype control mAb that had no effect (Figure 4G). Moreover, mAb 87B156 disrupted downstream MET signaling, as assessed by reduced ERK phosphorylation (p-ERK) (Supplemental Figure 6D). These results suggest that human polyreactive antibodies developed in patients with cancer, and those that cross-react with MET exhibit anti-cancer effects.

## Discussion

MET is a receptor tyrosine kinase that is related to many cancer types. While autoantibodies against oncogenes such as HER2 have been well studied for over 30 years, whether patients have autoantibodies against MET has not been reported. Our study describes the first isolation and characterization to our knowledge of mAbs that cross-react with MET in cancer patients. We show that out of the 100 patients analyzed, in 2 heterogeneous subcohorts of patients with breast and lung cancer, 13 had serological binding to MET.

Although over 90 mAbs are currently approved for treatment of cancer, none of them were isolated from patients. This is in contrast with antibodies against pathogens such as HIV-1, SARS-CoV-2, and Ebola, where clinically evaluated mAbs have been isolated from individuals who were infected with, and often recovered from, the same infection (40–43). This raises the question: can patients with cancer produce neutralizing antibodies against cancer cells? We succeeded in isolating autoantibodies from patients with cancer using the MET antigen receptor. However, comparing the multiple mAbs isolated from patients infected with pathogens to the autoantibodies described in the current study raises key differences between antibodies that target self versus foreign antigens. In contrast with naturally occurring antibodies against pathogens, MET-binding mAbs had low thermostability, implying a shorter half-life in the host. Moreover, the shift toward a non-IgG1 subclass suggests a more regulatory than activatory role for these antibodies. Polyreactivity was previously described for anti–HIV-1 antibodies from infected patients (44, 45). Such antibodies were linked to chronic HIV-1 infection and were strongly correlated with enhanced B cell expansion and antibody neutralization and breadth developing over several years of antibody-virus co-evolution from nonreactive germline B cell precursors. Similarly, MET-binding antibodies cloned during this study exhibited polyreactivity, and developed from nonreactive precursors. However, compared with anti–HIV-1 antibodies, the isolated autoantibodies were not clonally expanded and had low binding capabilities. Nevertheless, the successful isolation and characterization of autoantibodies from patients with cancer opens new avenues of understanding the self-directed B cell response during cancer.

In our cohort, MET binding in the serum was not correlated with MET expression in the tumors. This lack of correlation can be explained by the fact that the antibodies we detected were in fact not elicited by MET. These antibodies are most likely the by-product of immune dysregulation, a general trait that was reported for patients with cancer (21) that can result in generation of auto- and polyreactive B cells and antibodies. Moreover, MET expression is associated with poor survival (28, 46); while we observed significant positive correlation between MET binding in the serum and HR^+^ status, it is a positive prognosis. Taken together, our results point out that the capability of producing anti-MET autoantibodies in the periphery is not necessarily dependent on MET expression in the tumor and may be beneficial for the patients. This is supported by the fact that anti-MET mAbs were able to impact 3 different breast cancer cell lines. While additional research is needed to fully evaluate the anti-cancer potential of the autoantibodies isolated from patients, such an effect on tumor cells can represent the attempt of the host immune response to control the tumor.

## Methods

### Sex as a biological variable.

For patients with breast cancer, recruitment was limited to females due to the rarity of male patients with breast cancer, who constitute less than 1% of all cases. For patients with lung cancer, sex was not considered a biological variable. Serum samples from healthy donors were obtained without regard to sex.

### Study cohort.

Seventy-five patients with breast cancer and 25 patients with lung cancer were recruited from the Oncology Institute in Sheba Medical Center, Ramat Gan, Israel. The patients with breast cancer had metastatic disease or were undergoing neoadjuvant therapy, while the patients with lung cancer had metastatic disease or were receiving chemoradiation therapy. Detailed information about the patients’ disease is provided in Supplemental Table 1. Serum samples were initially collected from each patient to screen for anti-MET antibodies. Additional blood was drawn 2 to 4 times from patients with high anti-MET serum activity to provide a total of 150 mL of whole blood. Serum samples from healthy donors, used as controls, were obtained from the Israeli blood bank.

### Isolation of plasma and PBMCs from whole blood samples.

Plasma was isolated from a 5 mL blood sample that was centrifuged at 2000*g* for 10 minutes. The upper layer, containing the plasma, was carefully collected, aliquoted, and subsequently stored at –20°C. For PBMC isolation, whole blood was collected into K2EDTA tubes (BD Vacutainer) and subsequently diluted 3-fold with RPMI 1640 medium (Gibco). This mixture was then carefully layered onto Ficoll-Paque PLUS (Cytiva) in a 1:1 ratio for phase separation, following the manufacturer’s guidelines. The lymphocyte-rich buffy coat layer was collected into a new tube, washed, and resuspended in fetal bovine serum (FBS) supplemented with 10% DMSO, and then cryopreserved in liquid nitrogen.

### Expression of MET and SEMA.

A plasmid encoding the human MET was obtained in-house and the sequences corresponding to the extracellular portion of MET (EC-MET) and the ligand-binding domain, SEMA, were amplified using specific primers (TTATTCGTGCCACTCAATTTTTTGCGCTTCAAAGATGTCGTTCAGGCCGTGGTG, TGATCAGCGGTTTAAACTTAAGCTTTTATTCGTGCCACTCAATTTTTTGCG, GCATACATTAAACCAAAATGGCTACACACTGG, TGTGGAGCATACATTAAACCAAAATGGCTACACACTGGTTA, TAACCAGTGTGTAGCCATTTTGGTTTAATGTATGCTCCACA, CCAGTGTGTAGCCATTTTGGTTTAATGTATGC, CCCTCTAGACTCGAGCGGCCGCCCACCATGGAGACAGACACA, TGCTAGTGCCTCTTTACACTCGTCACCAGTGGAACCTGG, CCTGCAATCTACAAGGTTTTCCCA, GTTCAGGCCGTGGTGGTGGTGGTGGTGCACATAGGAGAATGTACTGTATTG, CACATAGGAGAATGTACTGTATTG, CCTGTAATAACAAGTATTTCGCCG, ACGGTAACTGAAGATGCTTGT, CCCATTGTCTATGAAATTCATCCA, TACATAAATGAGATCAAAGTATTTGGAAAG, CCTGTGTTTAAGCCTTTTGAAAAG, GTTCAGGCCGTGGTGGTGGTGGTGGTGTGTGAAATTCTGATCTGGTTGAAC, TGTGAAATTCTGATCTGGTTGAAC, AATGGCTTGGGCTGCAGA, CAGACAGATCTGTTGAGTCCATGT, GAGTGTAAAGAGGCACTAGCA, GTTCCAGGTTCCACTGGTGACGAGTGTAAAGAGGCACTAGCA, and CAATGGGATCTTCGTGATCTT; Integrated DNA Technologies). These sequences were cloned into pcDNA3.1 expression vectors. Each vector was designed with an N-terminal IgK signal peptide sequence (METDTLLLWVLLLWVPGSTGD) and incorporated 2 C-terminal tags for processing: a hexa-histidine sequence (HHHHHH, “His-tag”) for protein purification and a site-specific biotinylation sequence (GLNDIFEAQKIEWHE, “Avi-Tag”). These constructs were used for transient transfection of Expi293F cells by using the ExpiFectamine 293 Transfection Kit (Thermo Fisher Scientific). Seven days after transfection, the supernatant from the cultured cells was collected, passed through a 0.22 μm filter, and incubated with Ni^2+^-NTA agarose beads (Cytvia) for 2 hours at ambient temperature. Proteins were eluted with 250 mM imidazole, buffer exchanged to 1× PBS, aliquoted, and preserved at –80°C. Biotinylation of EC-MET protein was performed using the BirA biotin-protein ligase kit (Avidity LLC), in accordance with the manufacturer’s protocol.

### ELISA.

For serological screening by ELISA, high-binding 96-well ELISA plates (Corning, 9018) were prepared by coating with 5 μg/mL of MET, SEMA, or gp120 in 1× PBS and overnight incubation at 4°C. The next day, residual coating solution was discarded, and the wells were rinsed with a washing buffer composed of 1× PBS and 0.05% Tween 20 (Sigma-Aldrich). Subsequent blocking was performed at room temperature for 2 hours using 200 μL of blocking buffer containing 1× PBS, 3% BSA (MP Biomedicals), 20 mM EDTA, and 0.05% Tween 20. Plasma samples, diluted 1:50 in blocking buffer, or isolated IgG samples, diluted to 30 μg/mL in blocking buffer, were added to the plates and incubated for 1 hour at room temperature. All dilutions were chosen after titration experiments. After incubation, the plates underwent 3 wash cycles with the washing buffer. Secondary anti-IgG antibodies conjugated to horseradish peroxidase (HRP) (Jackson ImmunoResearch, 109-035-088) and diluted 1:5000 in blocking buffer, were then applied and allowed to incubate for 45 minutes at room temperature. Following an additional 4 washes, each well received 100 μL of TMB/E substrate (Millipore) and the absorbance was measured at 650 nm after 20 minutes using a BioTek 800 TS absorbance reader.

For mAb ELISA, high-binding 96-well ELISA plates were prepared by coating with 1 μg/mL of MET, SEMA, or gp120, following the same washing and blocking procedures as described above. mAbs were initially added at a concentration of 64 μg/mL, followed by 4 successive 4-fold dilutions in 1× PBS, and incubation for 1 hour at room temperature. The plates were then washed 3 times with washing buffer before addition of the secondary anti-IgG HRP-conjugated antibodies, as already described. After 4 additional washes, 100 μL of TMB/E substrate was applied to each well. The absorbance was measured at 650 nm after 20 minutes. For the polyreactivity ELISA, high-binding 96-well plates were prepared by coating each row with 1 μg/mL of MET, SEMA, HGF, BSA, ACE2, RBD, gp120, PSTS1, LPS, DNA, or insulin in 1× PBS overnight at 4°C, following the same washing and blocking procedures as described above. mAbs were added at a concentration of 16 μg/mL and incubated for 1 hour at room temperature. The plates were washed 3 times with washing buffer before adding the secondary anti-IgG HRP-conjugated antibodies, as detailed earlier. After 4 additional washes, 100 μL of TMB/E substrate was applied to each well. The absorbance was measured at 650 nm after 20 minutes.

For competition ELISA, high-binding 96-well ELISA plates were prepared by coating with 1 μg/mL of MET, following the same washing and blocking procedures as described above. HGF was initially added at 100, 1.56, 0.02, and 0 μg/mL and incubated for 1 hour at room temperature. The plates were then washed 3 times with washing buffer before addition of 15 μg of primary mAb for 10 minutes at room temperature, as described in the figure legends. A commercial anti-HGF mAb (5 μg/mL; Sigma-Aldrich, H7157) was included to assess HGF binding to MET. The plates were washed 3 times with washing buffer before adding the secondary anti-IgG HRP-conjugated antibodies, as detailed above. After 4 additional washes, 100 μL of TMB/E substrate was applied to each well. The absorbance was measured at 650 nm after 20 minutes.

All ELISAs were replicated at least 3 times.

### c-MET (SP44) immunostaining.

FFPE blocks were sectioned at 4 μm and a positive control was added on the right edge of the slides. Anti–c-MET (SP44) (Roche Tissue Diagnostics, 790-4430) immunostaining was calibrated on a Benchmark Ultra staining module (Roche Tissue Diagnostics). Slides were incubated at 60°C for 1 hour and were processed by a fully automated protocol. Briefly, after dewaxing and rehydration, sections were exposed to CC1 HIER pretreatment (Roche Tissue Diagnostics) for 64 minutes. Anti–c-MET (SP44) antibody (prediluted) was incubated at 37°C for 52 minutes. Detection was performed with an UltraView detection kit (Roche Tissue Diagnostics, 760-500) and amplification with an Amplification Kit (Roche Tissue Diagnostics, 760-080). Sections were counterstained with hematoxylin (Roche Tissue Diagnostics, 790-2208). At the end of the automated run, slides were dehydrated in graded ethanols (70%, 96%, and 100%), cleared in xylene, and film coverslipped. Stained sections were scanned and analyzed by a pathologist.

### Flow cytometry and direct single B cell sorting.

PBMCs were quickly thawed at 37°C and subsequently washed in 50 mL of RPMI 1640 medium. These cells were resuspended in FACS buffer that included 1% BSA and 2 mM EDTA in 1× PBS, and were stained with anti-CD19–VioBlue (Miltenyi Biotec, 130-120-031) and anti-IgG–FITC (Miltenyi Biotec, 130-118-340). All samples were then analyzed using a CytoFLEX S4 flow cytometer (Beckman Coulter).

For single B cell sorting, B cells were enriched with anti-CD19 magnetic beads (Miltenyi Biotec, 130-050-301) according to the manufacturer’s guidelines. These cells were resuspended in FACS buffer that included 1% BSA and 2 mM EDTA in 1× PBS, and were stained with anti-CD19–VioBlue (Miltenyi Biotec, 130-120-031), anti-IgG–FITC (Miltenyi Biotec, 130-118-340), and labeled MET via streptavidin-PE (Miltenyi Biotec, 130-106-790) and streptavidin-APC (Miltenyi Biotec, 130-106-792). Single CD19^+^IgG^+^MET^+^ cells were sorted to 96-well plates using a FACSAria III sorter (BD) as we previously described (35, 49, 50).

### Stimulation of B cells and indirect single B cell sorting.

PBMCs were quickly thawed at 37°C and subsequently washed in 50 mL of RPMI 1640 medium. B cells were then isolated using anti-CD19 magnetic beads (Miltenyi Biotec, 130-050-301) according to the manufacturer’s guidelines. B cells were cultured in complete RPMI medium supplemented with penicillin-streptomycin (Biowest) and 10 ng/mL human IL-15 (PeproTech) in a 96-well plate (Corning). After isolation, streptavidin microspheres (Bangs Laboratories) coated with biotinylated CpG ODN 2006 (InvivoGen) and biotinylated MET were introduced to the B cells. Three days after stimulation, the culture was supplemented with 50 ng/mL human IL-6 (PeproTech). After 3 additional days, B cells were harvested and stained with anti-CD27–FITC (Miltenyi Biotec, 30-113-629) and anti-CD38–APC (Miltenyi Biotec, 130-113-429). Single CD27^hi^CD38^hi^ cells were sorted to 96-well plates using a FACSAria III sorter as we previously described (32).

### Single B cell BCR sequencing.

Single B cells were sorted into a 96-well plate containing 4 μL of lysis buffer, which comprised 12 units of RNasin Ribonuclease Inhibitor (Promega, N2511), 10 mM DTT, and 0.5× PBS. Plates were immediately stored on dry ice prior to RNA reverse transcription and PCR amplification. For amplification, the lysed cells were thawed on ice. Ig genes were amplified as previously described (31, 35). cDNA synthesis was conducted with random hexamer primers (Invitrogen, 48190011) and SuperScript III Reverse Transcriptase (Invitrogen, 18080085). Antibody sequences for the gamma, kappa, and lambda chains were subsequently amplified through nested PCR, as previously described (35). The second-round PCR products were then purified, sequenced, and analyzed using IgBLAST (51).

### mAb cloning and production.

mAbs were cloned and produced as previously described (35). Briefly, the V(D)J regions of the mAbs were amplified from the first-round PCR products of sorted B cells and subsequently cloned into Ig expression vectors obtained from Michel C. Nussenzweig’s lab (The Rockefeller University, New York, New York, USA) (10, 52). For unmutated germline versions cloning, we designed compatible gBlocks and ordered the synthesis from Azenta. Cloned mAb vectors for both heavy and light chains were cotransfected into Expi293F cells (Thermo Fisher Scientific). Seven days after transfection, the cell supernatant was incubated with protein A–coated agarose beads (GE Life Sciences, 17519901). After several washing steps, antibodies were eluted from chromatography columns using 50 mM sodium phosphate (pH 3.0). Finally, antibodies were buffer exchanged into 1× PBS, aliquoted, and stored at –80°C.

### Immunofluorescence assay.

Breast carcinoma tissue sections, along with matched breast tissue slides (TissueArray, BR251f) or slides with breast carcinoma tissue sections and healthy normal tissues (TissueArray, BR1191) were initially baked for 60 minutes at 60°C. Following baking, slides underwent deparaffinization and antigen retrieval in Tris-EDTA buffer (pH 9) for 15 minutes. Subsequently, slides were blocked using 1% BSA for 120 minutes and then stained overnight at 4°C with biotinylated antibodies (69B287, 87B156, and mGO.53). The slides were then incubated with phalloidin conjugated to FITC (1:300; Sigma-Aldrich) and streptavidin conjugated to Alexa Fluor 647 (1:100; Miltenyi Biotec) for 2 hours at 4°C. Finally, slides were stained with DAPI (1:1000) and sealed using non-fluor mounting media (Prolong Gold Antifade, Invitrogen). Imaging was conducted using a TCS SP8 Confocal Microscope (Leica Microsystems).

### Thermal stability and cumulative volume measurements.

Particle sizing and thermal stability were evaluated employing dynamic light scattering (DLS) and differential scanning fluorimetry (DSF) with a Prometheus Panta instrument equipped with backreflection optics (NanoTemper Technologies). DLS analysis utilized light scattering at 405 nm with a photomultiplier tube in a backscatter orientation and with a solvent refractive index of 1.335. For thermal stability assessment, antibodies were analyzed by DSF (including backreflection) and DLS while subjected to thermal ramping from 25°C to 95°C. High-sensitivity capillaries (NanoTemper Technologies) and antibodies at a concentration of 1 mg/mL were utilized for all sizing and thermostability experiments. Data analysis was performed using the Prometheus Panta Control Software (NanoTemper Technologies).

### Cell lines.

The CAL-51 (DSMZ, ACC 302) cell line was cultured in Corning Dulbecco’s modified Eagle media (DMEM) supplemented with 10% FBS (Gibco), 1% penicillin/streptomycin (Biowest), 1% glutamine (Biowest), and 1% sodium pyruvate (Sartorius). The MDA-MB-468 (ATCC, HTB-132) and HCC70 (ATCC, CRL-2315) cell lines were cultured in RPMI 1640 supplemented with 10% FBS (Gibco), 1% penicillin/streptomycin (Biowest), 1% glutamine (Biowest), and 1% sodium pyruvate (Sartorius). Cell lines were continuously maintained in an incubator set at 37°C with 5% CO_2_ atmosphere and were regularly tested for mycoplasma contamination. Cell lines were acquired from the lab of Uri Ben-David (Department of Human Molecular Genetics & Biochemistry, Faculty of Medicine and Health Sciences, Tel Aviv University).

### Cancer cell line flow cytometry for mAb binding.

Cultured cells were harvested using Cell Dissociation Solution (Sartorius, 03-071-1B) and centrifuged at 400*g* for 5 minutes at 4°C. The resulting cell pellets were resuspended in FACS buffer and incubated for 30 minutes on ice with mAbs. Following a washing step, the cells were stained with anti–human IgG conjugated to APC (Miltenyi Biotec, 130-119-772). All samples were then analyzed using a CytoFLEX S4 flow cytometer (Beckman Coulter).

### Live cell imaging.

CAL-51, MDA-MB-468, or HCC70 cells (2 × 10^4^ each) were seeded in a 96-well plate (Corning) together with 500 nM of each mAb (87B156, 69B287, and mGO.53). The plates were then immediately placed in an Incucyte SX5 (Sartorius), and images were captured at 30-minute intervals. Data were analyzed using Incucyte analysis software and subsequently exported to GraphPad Prism software for further analysis. Live cell imaging was replicated 3 times.

For chemotactic cell migration and invasion experiments, 5000 CAL-51 cells were seeded in the upper chamber of each well in a 96-well chemotaxis microplate and treated with 100 μM mAbs (as described in the figure legends). EGF (1 μg/mL) was added to the lower chamber of all wells as a chemoattractant. Cell migration was monitored using live imaging, and the number of migrated CAL-51 cells was quantified.

### Western blot.

CAL-51 cells (2 × 10^4^) were seeded in a 96-well plate (Corning) and incubated with 500 nM of each mAb (87B156 and 69B287) for varying durations. The cells were then lysed in radioimmunoprecipitation assay buffer (150 mM sodium chloride, 1% Triton X-100, 0.5% sodium deoxycholate, 0.1 % SDS, 50 mM Tris, pH 8.0, protease inhibitors, and phosphatase inhibitors) and incubated on ice for 20 minutes with vortexing every 5 minutes. Lysates were then centrifuged for 15 minutes at 20,000*g* at 4°C. Determination of protein concentration was made using a Pierce BCA Protein Assay (Thermo Fisher Scientific, TS-23227). Ten micrograms from each lysate was boiled with SDS reducing sample buffer for 5 minutes, resolved by SDS-PAGE (Bio-Rad, 4568096), and transferred to Trans-Blot Turbo nitrocellulose membranes (Bio-Rad, 1704159). Following transfer, blots were blocked with 3% BSA in 1× PBS for 1 hour at room temperature and incubated with specific primary antibodies overnight at 4°C with gentle agitation. The following antibodies were used for biochemical studies: anti-ERK1 + ERK2 (Abcam, ab184699) and anti–p-ERK (Abcam, ab65142). HRP-conjugated anti-rabbit secondary antibodies (Jackson ImmunoResearch, 111-035-144) and ECL Reagent (Bio-Rad, 1705061) were used for detection.

### Statistics.

Statistical significance was determined using a *P*-value threshold of less than 0.05. Analysis was conducted using GraphPad Prism 10 software and the rstatix package in R (https://cran.r-project.org/web/packages/rstatix/index.html). All statistical tests applied were nonparametric, Mann-Whitney test for comparisons between 2 groups, the Kruskal-Wallis test for comparisons involving more than 2 groups, and Spearman’s test for correlation analyses. Unless indicated otherwise, corrections for multiple comparisons were performed with the false discovery rate (FDR) method and data are presented as all data points and median.

### Study approval.

All studies involving patient enrollment, sample collection, and clinical follow-up were approved by the Tel Aviv University Institutional Review Board (IRB) under protocol number 002606-3. Patients were recruited and followed at the Sheba Cancer Center, which received Helsinki Committee approval under number 5841-19-SMC. Written informed was obtained from all patients before first blood collection.

### Data availability.

The underlying data for the presented figures are available in the Supporting Data Values file. The MET-binding antibody sequences are available from the NCBI GenBank database (accession numbers: 1B217_HC_VDJ, PV363055; 1B217_LC_VDJ, PV363056; 1B233_HC_VDJ, PV363057; 1B233_LC_VDJ, PV363058; 23B307_HC_VDJ, PV363059; 23B307_LC_VDJ, PV363060; 69B253_HC_VDJ, PV363061; 69B253_LC_VDJ, PV363062; 69B287_HC_VDJ, PV363063; 69B287_LC_VDJ, PV363064; 87B156_HC_VDJ, PV363065; 87B156_LC_VDJ, PV363066; 92L204_HC_VDJ, PV363067; 92L204_LC_VDJ, PV363068; 92L205_HC_VDJ, PV363069; 92L205_LC_VDJ, PV363070; 92L236_HC_VDJ, PV363071; and 92L236_LC_VDJ, PV363072).

## Author contributions

NTF conceptualized the project, planned the experiments, supervised the experiments and data analyses, secured funding, and wrote the manuscript together with MN, MD, and ENGY. MN planned and performed the experiments, analyzed the data, prepared the figures, and wrote the manuscript. NBS analyzed the antibodies biochemically and performed the immunofluorescent staining, helped MN with the functional studies, analyzed the data, and prepared the figures. NTF, MN, and NBS had unrestricted access to all data. RY processed the blood samples and helped to analyze them. MM cloned the recombinant proteins and processed blood samples and helped to analyze them. MD, ENGY, and MBG recruited the patients with cancer and analyzed the patients’ data. DC, NBL, and IB performed the immunohistochemical assay for MET expression. IT provided the plasmid encoding human MET, advised on the first steps of the project, and contributed with fruitful discussions and ideas. All authors contributed to the discussion of the results and the preparation of the final manuscript.

## Supplementary Material

Supplemental data

Unedited blot and gel images

Supporting data values

## Figures and Tables

**Table 1:**
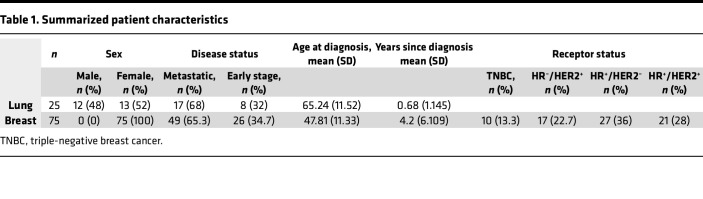
Summarized patient characteristics

## Version 1 05/22/2025

Electronic publication

## References

[1] Fridman WH (2021). B cells and cancer: to B or not to B?. J Exp Med.

[2] Paparoditis P, Shulman Z (2024). The tumor-driven antibody-mediated immune response in cancer. Curr Opin Immunol.

[3] Buckley CD (2015). Stromal cells in chronic inflammation and tertiary lymphoid organ formation. Annu Rev Immunol.

[4] Fridman WH (2022). B cells and tertiary lymphoid structures as determinants of tumour immune contexture and clinical outcome. Nat Rev Clin Oncol.

[5] Fridman WH (2023). Activation of B cells in tertiary lymphoid structures in cancer: anti-tumor or anti-self?. Semin Immunol.

[6] Wadle A (2006). Serological immune response to cancer testis antigens in patients with pancreatic cancer. Int J Cancer.

[7] DeFalco J (2018). Non-progressing cancer patients have persistent B cell responses expressing shared antibody paratopes that target public tumor antigens. Clin Immunol.

[8] Chauhan SK (2024). Peripheral immune cells in metastatic breast cancer patients display a systemic immunosuppressed signature consistent with chronic inflammation. NPJ Breast Cancer.

[9] Sautes-Fridman C (2019). Tertiary lymphoid structures in the era of cancer immunotherapy. Nat Rev Cancer.

[10] Wardemann H (2003). Predominant autoantibody production by early human B cell precursors. Science.

[11] Meffre E, Wardemann H (2008). B-cell tolerance checkpoints in health and autoimmunity. Curr Opin Immunol.

[12] Victora GD, Nussenzweig MC (2022). Germinal centers. Annu Rev Immunol.

[13] Elkon K, Casali P (2008). Nature and functions of autoantibodies. Nat Clin Pract Rheumatol.

[14] Gomez-Banuelos E (2023). Affinity maturation generates pathogenic antibodies with dual reactivity to DNase1L3 and dsDNA in systemic lupus erythematosus. Nat Commun.

[15] Zaenker P (2016). Autoantibody production in cancer--the humoral immune response toward autologous antigens in cancer patients. Autoimmun Rev.

[16] Shimada H (2003). Titration of serum p53 antibodies in 1,085 patients with various types of malignant tumors: a multiinstitutional analysis by the Japan p53 Antibody Research Group. Cancer.

[17] Oshima Y (2016). NY-ESO-1 autoantibody as a tumor-specific biomarker for esophageal cancer: screening in 1969 patients with various cancers. J Gastroenterol.

[18] Mazor RD (2022). Tumor-reactive antibodies evolve from non-binding and autoreactive precursors. Cell.

[19] Knutson KL (2016). Improved survival of HER2+ breast cancer patients treated with trastuzumab and chemotherapy is associated with host antibody immunity against the HER2 intracellular domain. Cancer Res.

[20] Popa X (2020). Anti-EGF antibodies as surrogate biomarkers of clinical efficacy in stage IIIB/IV non-small-cell lung cancer patients treated with an optimized CIMAvax-EGF vaccination schedule. Oncoimmunology.

[21] Crescioli S (2023). B cell profiles, antibody repertoire and reactivity reveal dysregulated responses with autoimmune features in melanoma. Nat Commun.

[22] Organ SL, Tsao MS (2011). An overview of the c-MET signaling pathway. Ther Adv Med Oncol.

[23] Uehara Y (1995). Placental defect and embryonic lethality in mice lacking hepatocyte growth factor/scatter factor. Nature.

[24] Duplaquet L (2018). The multiple paths towards MET receptor addiction in cancer. Oncogene.

[25] Lee YH (2014). Characterization of HGF/Met signaling in cell lines derived from urothelial carcinoma of the bladder. Cancers (Basel).

[26] Cooper CS (1992). The met oncogene: from detection by transfection to transmembrane receptor for hepatocyte growth factor. Oncogene.

[27] Malik R (2020). MET receptor in oncology: from biomarker to therapeutic target. Adv Cancer Res.

[28] Lengyel E (2005). C-Met overexpression in node-positive breast cancer identifies patients with poor clinical outcome independent of Her2/neu. Int J Cancer.

[29] Olivero M (1996). Overexpression and activation of hepatocyte growth factor/scatter factor in human non-small-cell lung carcinomas. Br J Cancer.

[30] Raghav KP (2012). cMET and phospho-cMET protein levels in breast cancers and survival outcomes. Clin Cancer Res.

[31] Tiller T (2008). Efficient generation of monoclonal antibodies from single human B cells by single cell RT-PCR and expression vector cloning. J Immunol Methods.

[32] Ben-Shalom N (2023). β2-adrenergic signaling promotes higher-affinity B cells and antibodies. Brain Behav Immun.

[33] Sanjuan Nandin I (2017). Novel in vitro booster vaccination to rapidly generate antigen-specific human monoclonal antibodies. J Exp Med.

[34] Vidarsson G (2014). IgG subclasses and allotypes: from structure to effector functions. Front Immunol.

[35] Mor M (2021). Multi-clonal SARS-CoV-2 neutralization by antibodies isolated from severe COVID-19 convalescent donors. PLoS Pathog.

[36] Notkins AL (2004). Polyreactivity of antibody molecules. Trends Immunol.

[37] Chen HT (2024). Human antibody polyreactivity is governed primarily by the heavy-chain complementarity-determining regions. Cell Rep.

[38] Uhlen M (2015). Proteomics. Tissue-based map of the human proteome. Science.

[39] Uhlen M (2005). A human protein atlas for normal and cancer tissues based on antibody proteomics. Mol Cell Proteomics.

[40] (2022). Human antibodies for viral infections. Annu Rev Immunol.

[41] Caskey M (2023). High volume subcutaneous delivery of long-acting HIV bNAbs. Lancet HIV.

[42] Gunst JD (2022). Early intervention with 3BNC117 and romidepsin at antiretroviral treatment initiation in people with HIV-1: a phase 1b/2a, randomized trial. Nat Med.

[43] Rosas-Umbert M (2022). Administration of broadly neutralizing anti-HIV-1 antibodies at ART initiation maintains long-term CD8^+^ T cell immunity. Nat Commun.

[44] Haynes BF (2005). Cardiolipin polyspecific autoreactivity in two broadly neutralizing HIV-1 antibodies. Science.

[45] Verkoczy L, Diaz M (2014). Autoreactivity in HIV-1 broadly neutralizing antibodies: implications for their function and induction by vaccination. Curr Opin HIV AIDS.

[46] Iovino F (2022). Expression of c-MET in estrogen receptor positive and HER2 negative resected breast cancer correlated with a poor prognosis. J Clin Med.

[47] Rabinowitz KM (2022). Anti-TNFα treatment impairs long-term immune responses to COVID-19 mRNA vaccine in patients with inflammatory bowel diseases. Vaccines (Basel).

[48] Hagin D (2021). Immunogenicity of Pfizer-BioNTech COVID-19 vaccine in patients with inborn errors of immunity. J Allergy Clin Immunol.

[49] Freund NT (2017). Coexistence of potent HIV-1 broadly neutralizing antibodies and antibody-sensitive viruses in a viremic controller. Sci Transl Med.

[50] Watson A (2021). Human antibodies targeting a Mycobacterium transporter protein mediate protection against tuberculosis. Nat Commun.

[51] Ye J (2013). IgBLAST: an immunoglobulin variable domain sequence analysis tool. Nucleic Acids Res.

[52] Scheid JF (2009). A method for identification of HIV gp140 binding memory B cells in human blood. J Immunol Methods.

