# Supplementary material for: Unique characteristics of autoantibodies targeting MET in patients with breast and lung cancer
Source: JCI Insight. 2025 May 22;10(10):e187392. doi: 10.1172/jci.insight.187392 (PMC12129041; doi:10.1172/jci.insight.187392)
Supplement: Supplemental data [file jciinsight-10-187392-s280.pdf]

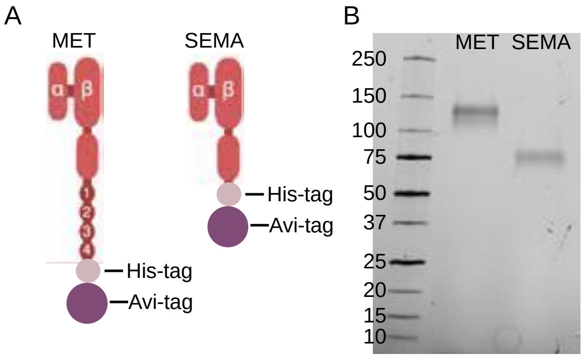

Supplementary Figure 1: MET and SEMA recombinant proteins. (A) Schematic illustration of MET and SEMA recombinant proteins and tags. (B) Protein SDS-PAGE showing recombinant MET and SEMA proteins after expression in Expi293 cells and affinity purification. The protein ladder is shown on the left.

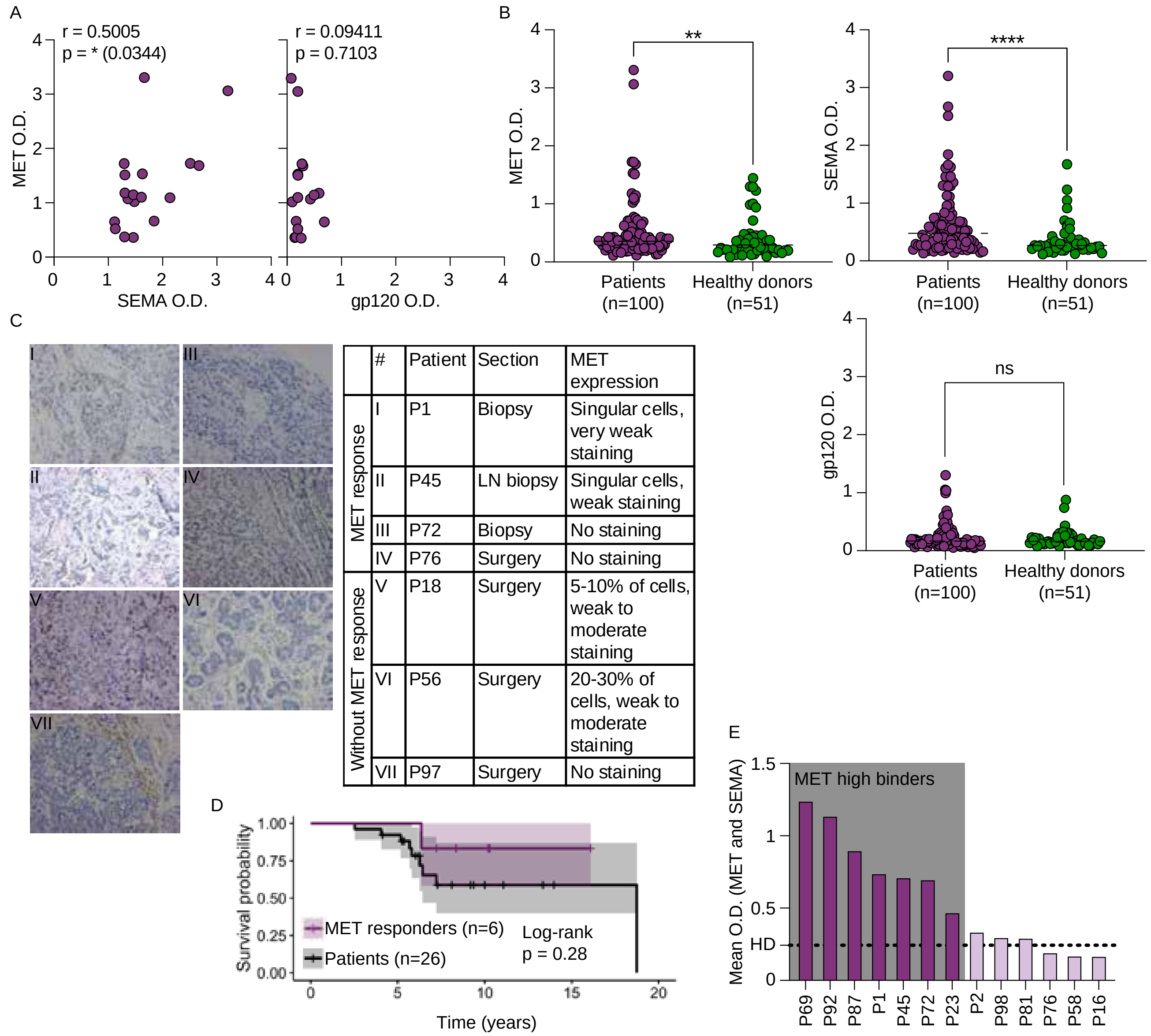

	#	Patient	Section	MET expression
MET response	I	P1	Biopsy	Singular cells, very weak staining
	II	P45	LN biopsy	Singular cells, weak staining
	III	P72	Biopsy	No staining
	IV	P76	Surgery	No staining
Without MET response	V	P18	Surgery	5-10% of cells, weak to moderate staining
	VI	P56	Surgery	20-30% of cells, weak to moderate staining
	VII	P97	Surgery	No staining

Supplementary Figure 2: Correlations and validation of anti-MET serological response. (A) Correlation plot between MET O.D.$_{650}$ values (y-axis) and the O.D.$_{650}$ values of either SEMA (left) or gp120 (right) of the 18 patients whose sera reacted to MET or SEMA. R and p-value are indicated, and were calculated with Spearman correlation. (B) MET, SEMA and gp120 O.D.$_{650}$ values of cancer patients (purple, n = 100) and non-cancer donors (green, n = 51). The p-value is calculated using the Kolmogorov-Smirnov test. (C) Slides of surgery or biopsy section from patients with serological MET response (P1, P45, P72, P76), stained for MET expression by immunohistochemistry, and a summarizing table. (D)

Kaplan-Meier survival plot of 32 HR+ metastatic breast cancer patients, comparing those who responded to MET (n=6) with those who did not respond to MET (n=26). (E) Bar graph representing the IgG binding, as the mean O.D. of MET and SEMA binding, for purified IgG based on ELISA. Samples IDs are indicated on the bottom part of the graph. The dashed line indicates the IgG score of the highest binding healthy donor.

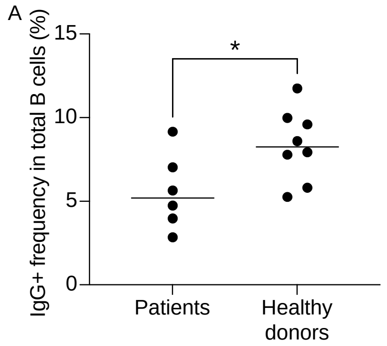

Supplementary Figure 3: Frequency of IgG+ B cells. Comparison between cancer patients (n = 6) and healthy donors (n = 8, data taken from previously published studies from the lab (51, 52)) of IgG+ cell frequencies out of total CD19 positive, measured by flow-cytometry. The p-value is calculated using the Mann-Whitney test.

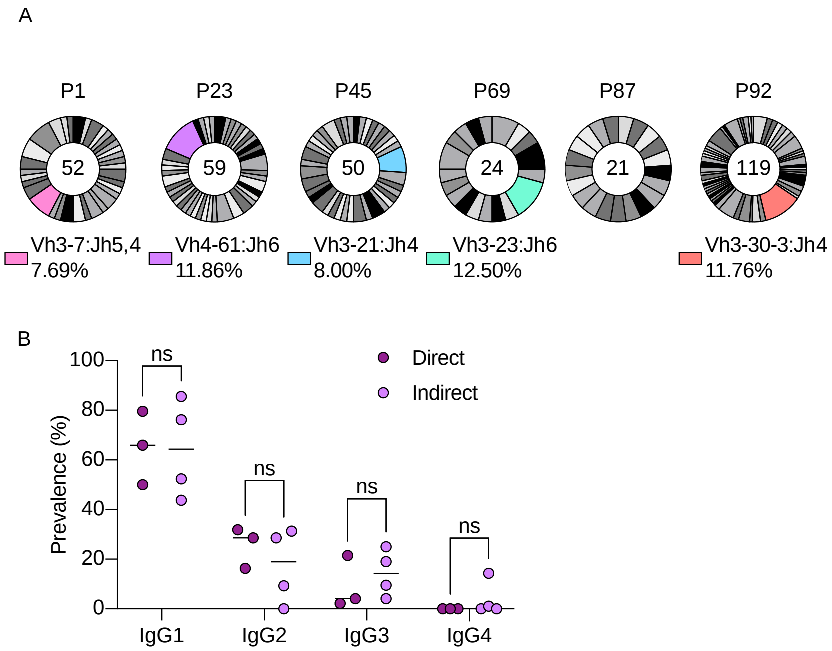

Supplementary Figure 4: IgH gene analysis in MET-enriched B cells. (A) Pie charts representing V_HJ_H combinations identified in each patient. The numbers in the middle of the pies represent the total sequences recovered, slices are proportional to each V_HJ_H combination. The most abundant V_HJ_H combination for each patient is colored. (B) IgG subclass prevalence of MET-binding sequences from direct sorting (n = 3) compared to sequences from indirect sorting (n = 4). The statistical analysis is calculated using multiple Mann-Whitney tests.

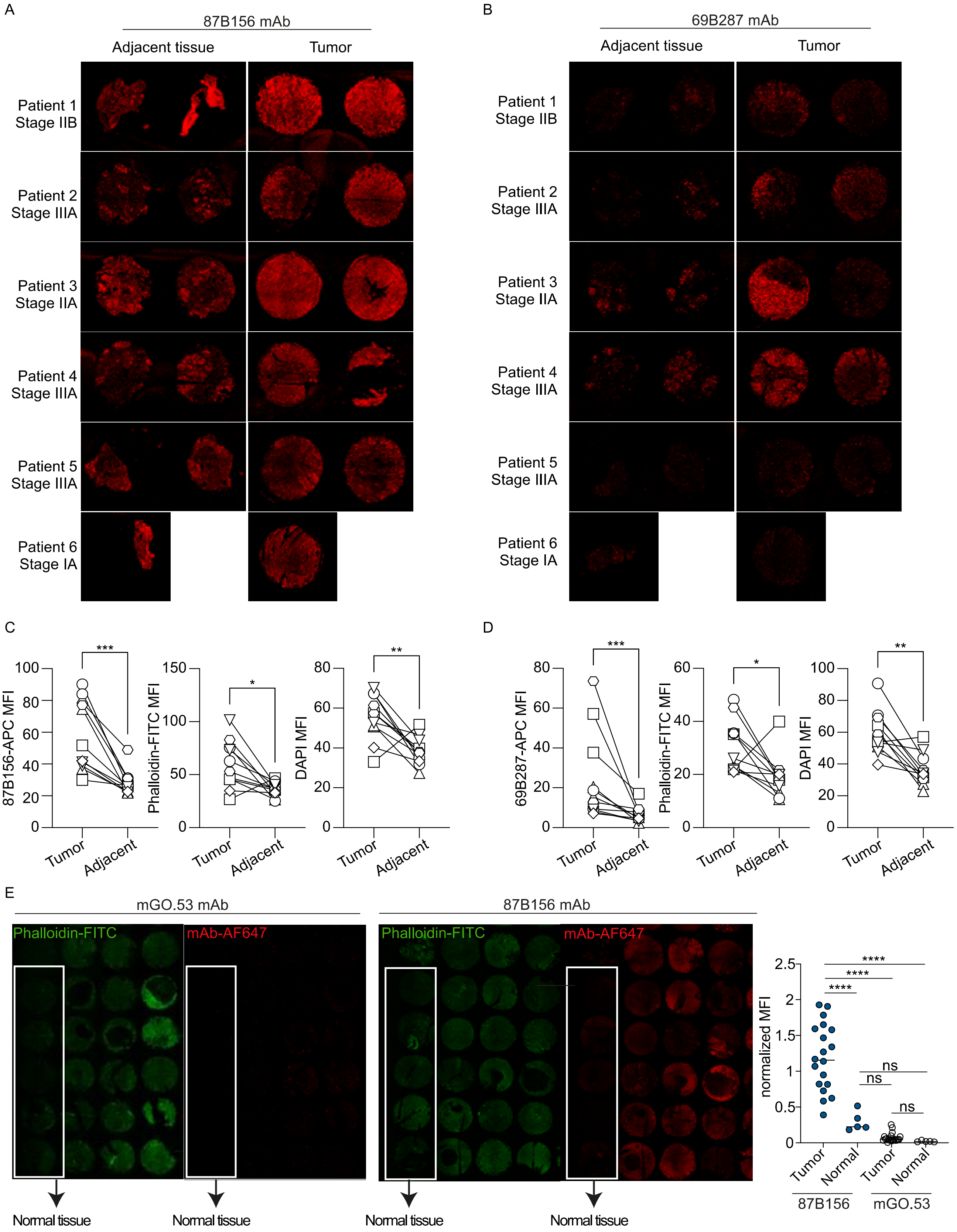
A
87B156 mAb
Adjacent tissue
Tumor
Patient 1 Stage IIB
Patient 2 Stage IIIA
Patient 3 Stage IIA
Patient 4 Stage IIIA
Patient 5 Stage IIIA
Patient 6 Stage IA
B
69B287 mAb
Adjacent tissue
Tumor
Patient 1 Stage IIB
Patient 2 Stage IIIA
Patient 3 Stage IIA
Patient 4 Stage IIIA
Patient 5 Stage IIIA
Patient 6 Stage IA
C
87B156-APC MFI
Phalloidin-FITC MFI
DAPI MFI
Tumor
Adjacent
***
*
**
D
69B287-APC MFI
Phalloidin-FITC MFI
DAPI MFI
Tumor
Adjacent
***
*
**
E
mGO.53 mAb
87B156 mAb
Phalloidin-FITC
mAb-AF647
Normal tissue
normalized MFI
Tumor
Normal
87B156
mGO.53
****
ns

Supplementary Figure 5: Binding of 69B287 and 87B156 mAbs to tumor tissues. (A-B) images of tumor tissue (n = 11) and healthy adjacent tissue (n = 11) stained mAb conjugated to Alexa Fluor 647 (A) 87B156 and (B) 69B287. (C-D) quantification of raw MFI acquired for mAb, phalloidin, and DAPI for slides stained with (C) 87B156 and (D) 69B287. E. Images of tumor tissues and healthy tissues stained with phalloidin and isotype control mAb, mGO.53 (left) and 87B156 mAb (right). (D) Quantification and normalization of mGO.53 and 87B156 mAbs to phalloidin between tumor and healthy tissues. p-value is calculated by one-way analysis of variance (ANOVA) with Tukey's multiple comparisons post-test.

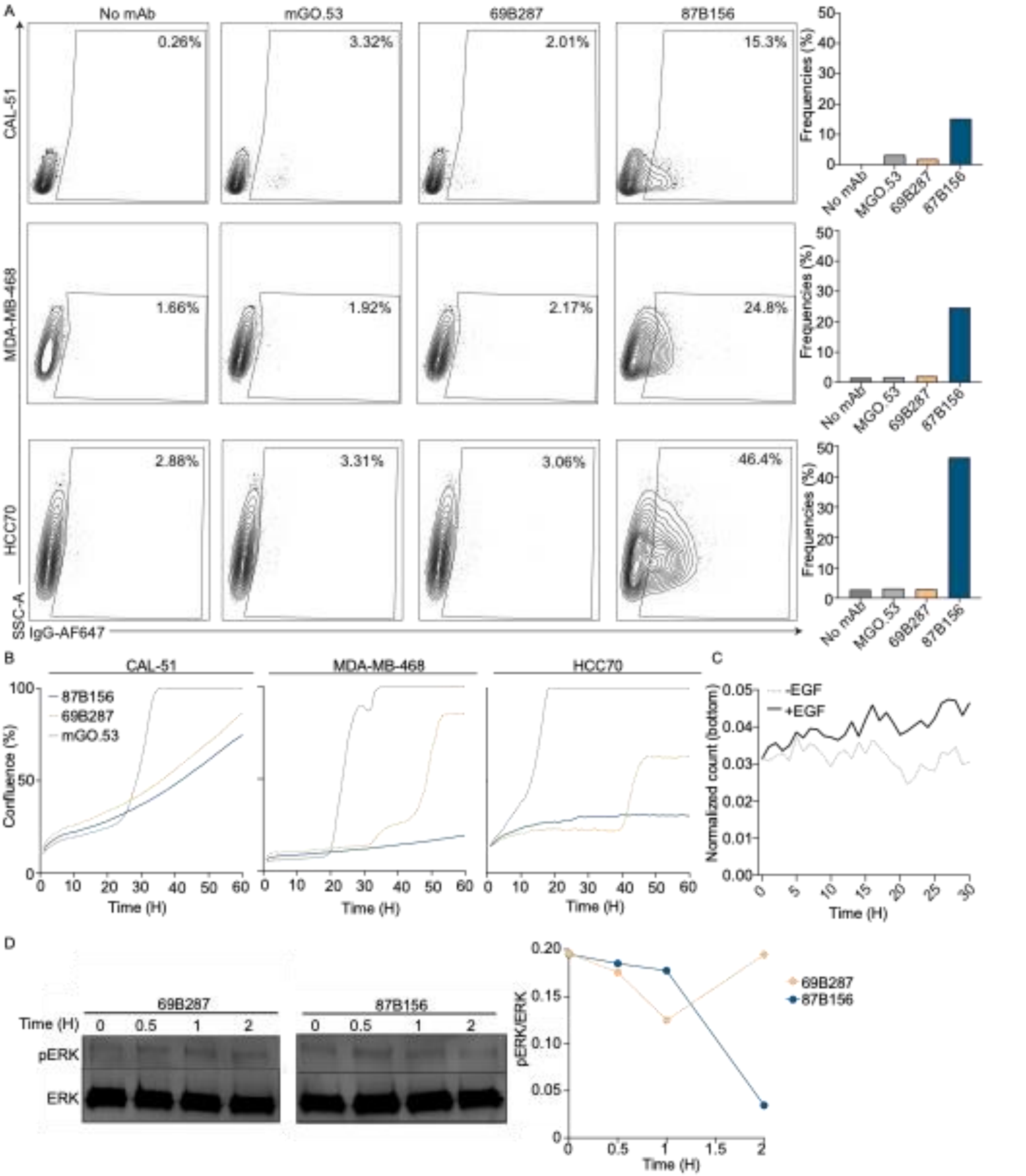

Supplementary Figure 6: Binding of 69B287 and 87B156 mAbs to cancer cell lines. (A) Flow cytometry plots (left) and the summarized frequencies (right) showing the binding of mGO.53, 69B287,

and 87B156 to CAL-51 (top), MDA-MB-468 (middle) and HCC70 (bottom) breast cancer cell lines. (B) Confluence measured by Incucyte of CAL-51, MDA-MB-468 and HCC70 breast cancer cell lines over time, incubated with each mAb (87B156, 69B287, and mGO.53), with 4-6 repetitions for each condition. (C) Quantification of chemotactic cell migration and invasion. A total of 5,000 CAL-51 cells were seeded in the upper chamber of each well in a 96-well chemotaxis microplate. EGF (1 µg/mL) was either included in or excluded from the lower chamber as a chemoattractant. Cell migration was monitored via live imaging, and the number of migrated CAL-51 cells was quantified. Migration counts were normalized to the initial cell number, with 3 repetitions for each condition. (D) Left panel: Western blot analysis showing pERK and ERK levels in CAL-51 breast cancer cells treated with mAbs 69B287 and 87B156 at the indicated time points. Right panel: Graph depicting the fold change in pERK levels over time, normalized to total ERK levels. The western blot was repeated twice.

743 **Supplementary Table 1 : Detailed Patient characteristics**

Patient	Cancer type	Cancer subtype	Gender	Age at diagnosis	Years since diagnosis	Disease status	Anti-MET response
**P1**	Breast	HR+HER2-	Female	52	0	Early stage	Positive
**P2**	Breast	HR+HER2+	Female	43	11	Metastatic	Negative
**P3**	lung	Other	Male	69	2	Metastatic	Negative
**P4**	lung	Adenocarcinoma	Female	72	0	Metastatic	Negative
**P5**	Breast	HR+HER2-	Female	60	0	Metastatic	Negative
**P6**	Lung	Adenocarcinoma	Female	64	0	Metastatic	Negative
**P7**	Breast	TNBC	Female	55	0	Early stage	Negative
**P8**	Breast	HR-HER2+	Female	47	11	Metastatic	Negative
**P9**	Breast	HR-HER2+	Female	62	0	Metastatic	Negative
**P10**	Breast	HR+HER2-	Female	43	5	Metastatic	Negative
**P11**	Lung	Adenocarcinoma	Male	66	1	Metastatic	Negative
**P12**	Breast	HR+HER2-	Female	39	0	Metastatic	Negative
**P13**	Breast	HR+HER2-	Female	54	23	Metastatic	Negative
**P14**	Breast	HR+HER2-	Female	62	3	Metastatic	Negative
**P15**	Lung	Adenocarcinoma	Female	81	1	Metastatic	Negative
**P16**	Breast	HR+HER2+	Female	45	0	Early stage	Negative
**P17**	Breast	HR+HER2-	Female	35	0	Early stage	Negative
**P18**	Lung	Other	Female	77	1	Metastatic	Negative
**P19**	Breast	TNBC	Female	59	0	Early stage	Negative
**P20**	Lung	Other	Female	72	0	Metastatic	Negative
**P21**	Breast	HR+HER2-	Female	53	13	Metastatic	Negative
**P22**	Lung	Adenocarcinoma	Male	70	0	Metastatic	Negative
**P23**	Breast	HR+HER2+	Female	63	0	Metastatic	Positive
**P24**	Breast	HR+HER2+	Female	56	0	Early stage	Negative
**P25**	Breast	HR-HER2+	Female	37	1	Early stage	Negative
**P26**	Lung	Adenocarcinoma	Female	68	0	Early stage	Negative
**P27**	Breast	HR-HER2+	Female	48	3	Metastatic	Negative
**P28**	Breast	HR+HER2-	Female	35	0	Early stage	Negative
**P29**	Breast	HR-HER2+	Female	30	3	Metastatic	Negative
**P30**	Lung	Other	Female	59	0	Early stage	Negative
**P31**	Breast	HR+HER2+	Female	44	4	Metastatic	Negative
**P32**	Breast	TNBC	Female	67	1	Metastatic	Negative
**P33**	Breast	HR-HER2+	Female	38	0	Metastatic	Negative
**P34**	Lung	Adenocarcinoma	Female	71	1	Metastatic	Negative
**P35**	Breast	HR+HER2+	Female	69	2	Metastatic	Negative
**P36**	Breast	TNBC	Female	47	1	Early stage	Negative
**P37**	Breast	HR+HER2-	Female	49	1	Early stage	Negative
**P38**	Breast	HR+HER2+	Female	45	0	Early stage	Negative
**P39**	Breast	HR-HER2+	Female	45	0	Early stage	Negative
**P40**	Breast	HR+HER2+	Female	43	0	Metastatic	Negative
**P41**	Lung	Squamous cell carcinoma	Male	61	0	Metastatic	Negative
**P42**	Breast	HR+HER2-	Female	67	1	Metastatic	Negative
**P43**	Breast	HR-HER2+	Female	36	0	Early stage	Negative
**P44**	Lung	Other	Female	67	1	Early stage	Negative

**P45**	Breast	HR+HER2-	Female	32	0	Early stage	Positive
**P46**	Lung	Adenocarcinoma	Female	78	0	Early stage	Negative
**P47**	Breast	HR-HER2+	Female	81	4	Metastatic	Negative
**P48**	Breast	HR-HER2+	Female	40	0	Early stage	Negative
**P49**	Breast	HR+HER2-	Female	41	8	Metastatic	Negative
**P50**	Breast	HR+HER2-	Female	50	0	Metastatic	Negative
**P51**	Breast	HR-HER2+	Female	76	1	Metastatic	Negative
**P52**	Breast	HR+HER2+	Female	43	2	Metastatic	Negative
**P53**	Breast	HR+HER2-	Female	45	0	Early stage	Negative
**P54**	Lung	Adenocarcinoma	Male	74	0	Metastatic	Negative
**P55**	Breast	TNBC	Female	40	0	Metastatic	Negative
**P56**	Breast	HR+HER2+	Female	40	0	Early stage	Negative
**P57**	Breast	HR+HER2-	Female	46	13	Metastatic	Negative
**P58**	Lung	Other	Female	72	0	Early stage	Positive
**P59**	Breast	TNBC	Female	35	0	Early stage	Negative
**P60**	Breast	HR+HER2+	Female	38	3	Metastatic	Negative
**P61**	Lung	Squamous cell carcinoma	Male	76	0	Early stage	Negative
**P62**	Breast	HR+HER2+	Female	68	0	Early stage	Negative
**P63**	Lung	Adenocarcinoma	Male	45	2	Metastatic	Positive
**P64**	Lung	Adenocarcinoma	Male	54	5	Metastatic	Positive
**P65**	Breast	TNBC	Female	26	2	Metastatic	Negative
**P66**	Lung	Squamous cell carcinoma	Male	62	0	Metastatic	Negative
**P67**	Breast	HR+HER2-	Female	63	14	Metastatic	Negative
**P68**	Breast	HR+HER2-	Female	46	17	Metastatic	Negative
**P69**	Breast	HR+HER2-	Female	46	15	Metastatic	Positive
**P70**	Lung	Squamous cell carcinoma	Female	64	2	Metastatic	Negative
**P71**	Breast	HR+HER2-	Female	45	7	Metastatic	Negative
**P72**	Lung	Other	Male	60	1	Early stage	Positive
**P73**	Breast	HR+HER2-	Female	46	26	Metastatic	Negative
**P74**	Breast	HR+HER2+	Female	38	4	Metastatic	Positive
**P75**	Breast	TNBC	Female	47	2	Metastatic	Negative
**P76**	Breast	HR+HER2+	Female	45	6	Metastatic	Positive
**P77**	Breast	HR+HER2-	Female	48	0	Early stage	Negative
**P78**	Breast	HR+HER2-	Female	69	3	Metastatic	Negative
**P79**	Breast	TNBC	Female	42	1	Early stage	Negative
**P80**	Breast	HR+HER2-	Female	47	4	Metastatic	Negative
**P81**	Lung	Other	Male	65	0	Metastatic	Negative
**P82**	Breast	HR-HER2+	Female	40	0	Early stage	Negative
**P83**	Breast	HR+HER2+	Female	39	14	Metastatic	Negative
**P84**	Breast	HR+HER2+	Female	44	12	Metastatic	Negative
**P85**	Breast	HR+HER2+	Female	54	5	Metastatic	Negative
**P86**	Breast	HR-HER2+	Female	53	16	Metastatic	Negative
**P87**	Breast	HR+HER2+	Female	50	6	Metastatic	Positive
**P88**	Breast	HR+HER2+	Female	51	0	Metastatic	Negative
**P89**	Breast	HR-HER2+	Female	46	0	Early stage	Negative
**P90**	Breast	HR-HER2+	Female	31	0	Metastatic	Negative
**P91**	Breast	HR-HER2+	Female	41	0	Metastatic	Negative
**P92**	Lung	Other	Male	58	0	Metastatic	Positive
**P93**	Breast	TNBC	Female	34	0	Early stage	Negative

**P94**	Breast	HR+HER2-	Female	42	0	Early stage	Negative
**P95**	Breast	HR+HER2+	Female	53	15	Metastatic	Negative
**P96**	Breast	HR+HER2-	Female	60	16	Metastatic	Negative
**P97**	Breast	HR-HER2+	Female	26	0	Early stage	Negative
**P98**	Breast	HR+HER2+	Female	63	5	Metastatic	Positive
**P99**	Lung	Other	Female	68	0	Early stage	Negative
**P100**	Breast	HR+HER2-	Female	47	11	Metastatic	Negative

Supplementary Table 2 : Detailed Patient characteristics

Patient	Sorted - Direct	Recoverd HC - Direct	Recoverd LC - Direct	Sorted - Indirect	Recoverd HC - Indirect	Recoverd LC - Indirect
**P1**	48	14	31	84	38	40
**P23**	108	59	89			
**P45**	211	50	46			
**P69**	69	0	0	134	24	68
**P87**				120	21	42
**P92**				192	119	114
