# Supplementary material for: Unique characteristics of autoantibodies targeting MET in patients with breast and lung cancer
Source: JCI Insight. 2025 May 22;10(10):e187392. doi: 10.1172/jci.insight.187392 (PMC12129041; doi:10.1172/jci.insight.187392)

Supplementary Figure 1: MET and SEMA recombinant proteins. (B) Protein SDS-PAGE showing recombinant MET and SEMA proteins after expression in Expi293 cells and affinity purification.

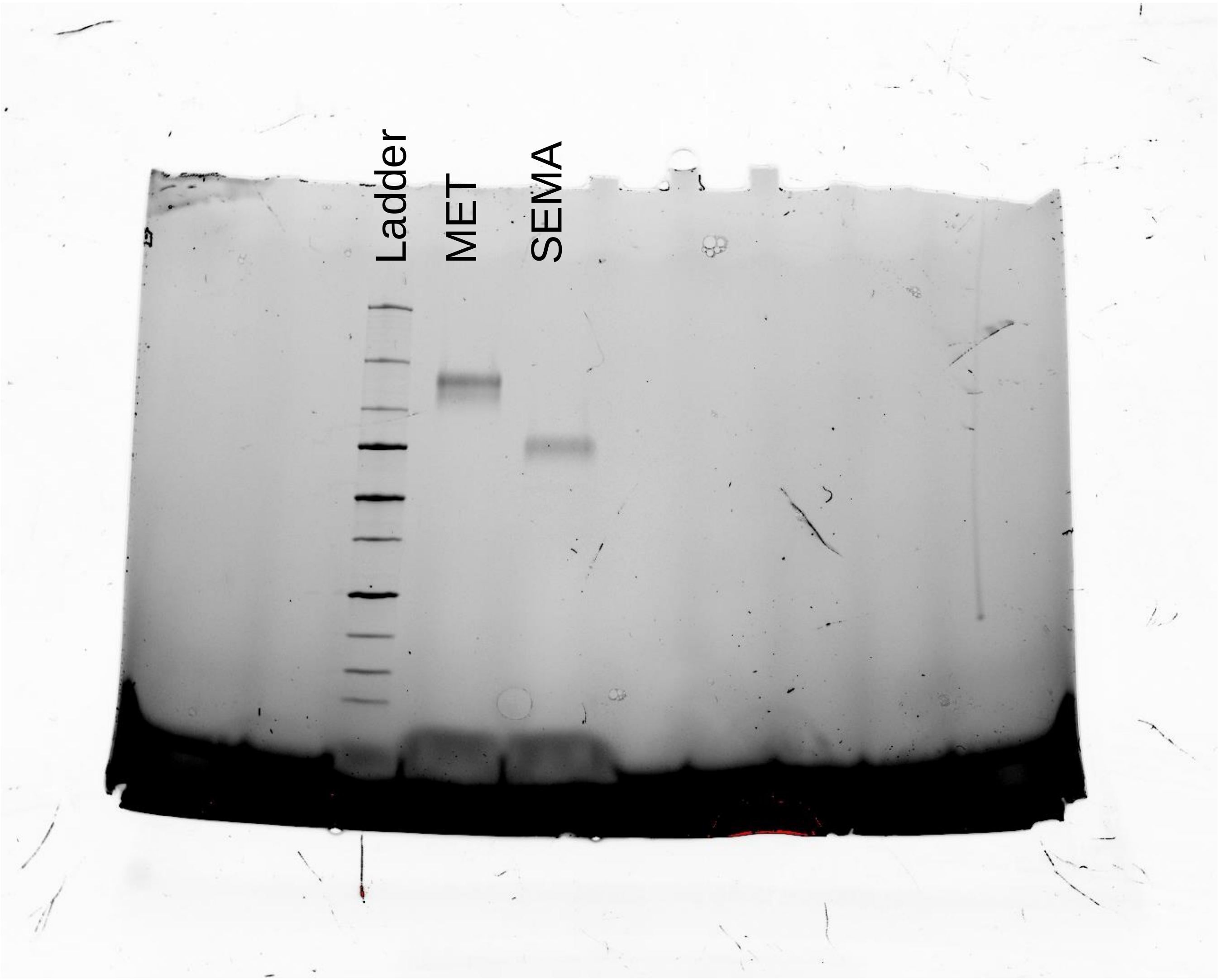

Supplementary Figure 6: Binding of 69B287 and 87B156 mAbs to cancer cell lines. (D) Left panel: Western blot analysis showing pERK and ERK levels in CAL-51 breast cancer cells treated with mAbs 69B287 and 87B156 at the indicated time points.

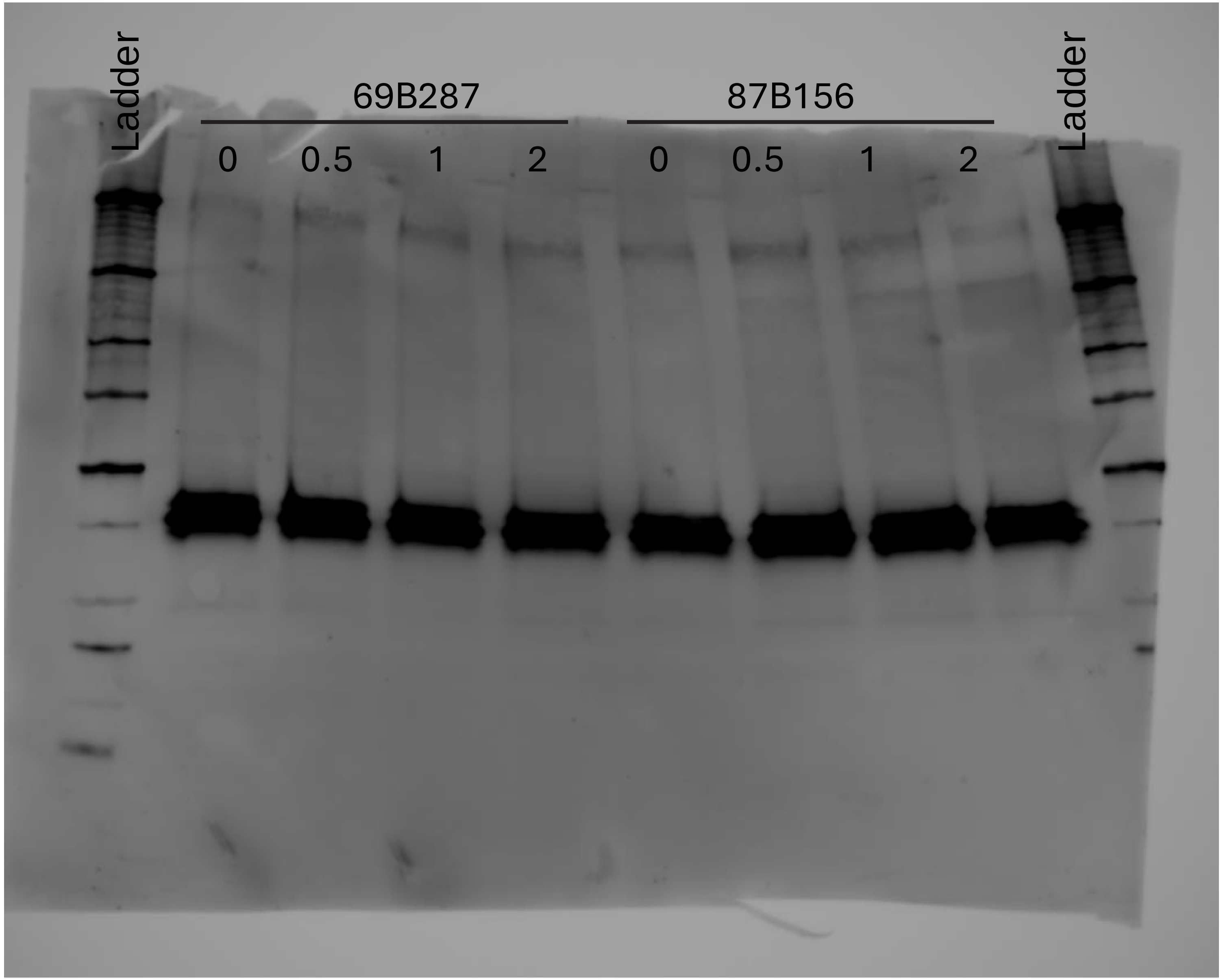

Supplement: Unedited blot and gel images [file jciinsight-10-187392-s281.pdf]
